# Computer Vision for 3D Perception and Applications

**DOI:** 10.3390/s21123944

**Published:** 2021-06-08

**Authors:** Matteo Poggi, Thomas B. Moeslund

**Affiliations:** 1 Department of Computer Science and Engineering (DISI), University of Bologna, 40136 Bologna, Italy; 2 Visual Analysis and Perception (VAP) Lab, Aalborg University, Rendsburggade 14, 9000 Aalborg, Denmark; tbm@create.aau.dk

Effective 3D perception of an observed scene greatly enriches the knowledge about the surrounding environment and is crucial to effectively develop high-level applications for various purposes. Pivotal to 3D perception is the acquisition/estimation of reliable depth information—a task for which several technologies exist, ranging from active sensors (e.g., Time-of-Flight devices) to passive cameras, these latter coupled with a variety of different techniques allowing for depth estimation from images (stereo matching [1], structure from motion [2], and more). From an accurate reconstruction of the surrounding 3D scene, several complex problems can be addressed, such as autonomous navigation and localization, tracking, surveillance, recognition, robotics, interaction with other agents, and manipulation of the sensed environment. Additionally, in this field, recent advances in deep learning have been rapidly established and are gaining momentum.

The Special Issue of *Sensors* entitled “Computer Vision for 3D Perception and Applications” aims to provide new insights into a variety of applications building upon 3D knowledge of the scene, varying from the earliest stage of the 3D acquisition process to the higher-level tasks tackled over 3D data. For this purpose, from the vast number of manuscripts received, 11 original and high-quality manuscripts were selected for inclusion in this Special Issue. Each manuscript was reviewed by prestigious researchers specializing in the same topics as the articles and was subjected to up to three rounds of peer review. Each of the articles presents advances in research in the field of computer vision and 3D understanding, applied to a range of interesting and relevant application domains. We hope that this Special Issue provides an inspirational collection of ideas, techniques, and design strategies for 3D computer vision applications, which will continue to stimulate further research within this state-of-the-art domain.

Among the several tasks concerning 3D computer vision, the research concerning 3D descriptors has always attracted much attention from the community. In the first paper, entitled “PPTFH: Robust Local Descriptor Based on Point-Pair Transformation Features for 3D Surface Matching” [3], a novel 3D local surface descriptor is proposed. In particular, it is designed to deal with well-known issues such as noise, mesh decimation, clutter, and occlusions often occurring in real scenes, in order to achieve better distinctiveness and robustness and thus ease the 3D surface matching task.

The second paper, “Simulation Study of a Frame-Based Motion Correction Algorithm for Positron Emission Imaging” [4], deals with Positron Emission Tomography (PET), a non-invasive imaging modality that uses radiotracers to measure changes in metabolic processes, and with image degradation due to involuntary motions of the patients inside the scanner. Specifically, a simulation study is conducted to measure the performance of an image motion correction method based on a frame-based algorithm. The experiments show that the studied method minimizes intra-frame motion and improves the signal intensity with respect to the background compared to other approaches in the literature.

Time-of-Flight (ToF) sensors represent a popular choice among active depth sensors, and the Multi-Path Interference (MPI) effect is tackled in the third paper, “Deep Learning for Transient Image Reconstruction from ToF Data” [5]. Specifically, MPI artefacts are caused by the multiple reflections of light occurring in the sensed scene. To reduce the effect of this phenomenon, a deep learning framework is proposed, estimating the structure of the time-dependent impulse response of the scene and thus recovering a depth map from it, with a reduced quantity of MPI artefacts.

The fourth paper, “Combining Augmented Reality and 3D Printing to Improve Surgical Workflows in Orthopedic Oncology: Smartphone Application and Clinical Evaluation” [6], explores the field of medical imaging and introduces an innovative solution for orthopedic oncological surgery, combining 3D printing and Augmented Reality (AR). The surgeons are aided by a smartphone application using AR to display the patient’s anatomy on 3D-printed models, which eases the different steps of the surgical workflow, from planning to patient communication and surgical intervention itself.

Some high-level applications such as human pose or joint position estimation, which are traditionally performed on standard RGB images, can benefit from 3D sensing as well. As shown in the fifth paper, “HRDepthNet: Depth Image-Based Marker-Less Tracking of Body Joints” [7], processing depth information is beneficial for the estimation of human pose. Indeed, by retraining a state-of-the-art pose estimation network (HRNet) on RGB plus depth images allows more accurate estimation human poses with respect to using standard images only, confirming that 3D knowledge can be meaningful for the performance of several tasks.

The sixth paper, “Surface Reconstruction from Structured Light Images Using Differentiable Rendering” [8], deals with the acquisition of 3D data, specifically from structured light images. Indeed, most acquisition techniques usually acquire sparse representations of the collected scene, referred to as point clouds, while a dense reconstruction in the form of a 3D mesh is usually obtained with algorithms. In this work, a single-step procedure relying on differentiable rendering allows for sharp reconstructions of continuous 3D surfaces, resulting in robustness to image noise.

The seventh paper, “A Systematic Comparison of Depth Map Representations for Face Recognition” [9], proposes a study about the generalization capabilities and performance of deep face recognition methods that process depth data, inquiring about how the different sensing technologies, the quality of the perceived depth map, and the acquisition distance affect the performance of the face recognition task. This study reveals that methods based on normal images and point clouds result in greater robustness and better generalization with respect to other 2D and 3D alternatives.

As for most problems in computer vision, the state-of-the-art solutions concerning 3D descriptors are nowadays based on deep learning as well, accompanied by the need for annotated data. The eight paper of this Special Issue, entitled “Self-Supervised Point Set Local Descriptors for Point Cloud Registration” [10], proposes a self-supervised pipeline to learn a 3D local registration descriptor, allowing a reduction in the burden required to train a learning-based detector since it does not require data annotation; nonetheless, it can still achieve performance that is superior with respect to a fully supervised counterpart.

In parallel to active sensors, depth estimation from standard images has always represented an attractive and low-cost alternative. The ninth paper, “Real-Time Single Image Depth Perception in the Wild with Handheld Devices” [11], shows that single-image depth estimation based on deep learning is now a mature technology in terms of both efficiency and accuracy/generalization to a variety of image content. In particular, a properly designed deep network can be deployed on consumer smartphones and yet achieve real-time performance.

In the tenth paper, entitled “Semantic Extraction of Permanent Structures for the Reconstruction of Building Interiors from Point Clouds” [12], indoor 3D reconstruction is addressed at a higher level by means of a semantic understanding of the scene. Specifically, in order to prevent reconstruction errors due to clutter or occlusions, semantic scene completion is performed in order to extract permanent structures such as walls, floors, and ceilings, which are assumed to be planar. If correctly identified, these structures can ignore noise and artefacts in the 3D point cloud and improve 3D reconstructions.

Finally, as several depth sensing technologies exist with different strengths and weaknesses, the choice of one among the others represents a key factor in the deployment of effective solutions to higher-level tasks, particularly when automating tasks that are usually performed by human operators. The last paper of this Special Issue, entitled “3D Sensors for Sewer Inspection: A Quantitative Review and Analysis” [13], a study of different depth perception approaches—ToF, passive stereo, and active stereo—is conducted to identify the solution that is best-suited for sewer inspection. From this study, ToF-based acquisitions are found to be superior with respect to active and passive stereo ones.

In summary, there exist a variety of tasks for which 3D knowledge is a precious source of information to properly tackle a problem. As suggested by the title of this Special Issue, both the research lines focusing on depth and geometry perception and those that build upon already collected 3D data are experiencing rapid growth. By reviewing the high-quality papers published in this Special Issue, we can see how deep learning is nowadays widespread in both perception and application fields. Moreover, the ever-increasing diffusion of low-cost active sensors, which are reaching even consumer devices such as smartphones and tablets, further demonstrates the increasing interest of the research community in developing new solutions, creating new opportunities for practitioners and end-users. Consequently, we foresee the expansion of many 3D applications. 
